# Predicting speculation: a simple disambiguation approach to hedge detection in biomedical literature

**DOI:** 10.1186/2041-1480-2-S5-S7

**Published:** 2011-10-06

**Authors:** Erik Velldal

**Affiliations:** 1 Department of Informatics, University of Oslo, PO Box 1080 Blindern, 0316 Oslo, Norway

## Abstract

**Background:**

This paper presents a novel approach to the problem of *hedge detection*, which involves identifying so-called hedge cues for labeling sentences as certain or uncertain. This is the classification problem for Task 1 of the CoNLL-2010 Shared Task, which focuses on hedging in the biomedical domain. We here propose to view hedge detection as a simple disambiguation problem, restricted to words that have previously been observed as hedge cues. As the feature space for the classifier is still very large, we also perform experiments with dimensionality reduction using the method of *random indexing*.

**Results:**

The SVM-based classifiers developed in this paper achieves the best published results so far for sentence-level uncertainty prediction on the CoNLL-2010 Shared Task test data. We also show that the technique of random indexing can be successfully applied for reducing the dimensionality of the original feature space by several orders of magnitude, without sacrificing classifier performance.

**Conclusions:**

This paper introduces a simplified approach to detecting speculation or uncertainty in text, focusing on the biomedical domain. Evaluated at the sentence-level, our SVM-based classifiers achieve the best published results so far. We also show that the feature space can be aggressively compressed using random indexing while still maintaining comparable classifier performance.

## Background

### Introduction: hedge detection

The problem of hedge detection refers to the task of identifying *uncertainty* or *speculation* in text. Being the topic of several recent shared tasks and dedicated workshops this is a problem that is receiving increased interest within the fields of NLP and biomedical text mining (for example, hedging played a central role in the shared tasks of both BioNLP 2009 and CoNLL 2010, as well as the NeSp-NLP 2010 workshop). In terms of practical motivation, hedge detection is particularly useful in relation to information extraction tasks, where the ability to distinguish between factual and uncertain information can be of vital importance.

The topic of the Shared Task at the 2010 Conference for Natural Language Learning (CoNLL) is hedge detection for the domain of biomedical research literature [1]. The task is defined for two levels of analysis: While Task 1 is described as *learning to detect sentences containing uncertainty*, the object of Task 2 is *learning to resolve the in-sentence scope of hedge cues.* The focus of the present paper is only on Task 1.

A hedge cue is here taken to mean the words or phrases that signal the attitude of uncertainty or speculation. As noted by Farkas et al. [1], most hedge cues typically fall in the following categories; adjectives or adverbs (*probable*, *likely*, *possible*, *unsure*, etc.), auxiliaries (*may*, *might*, *could*, etc.), conjunctions (*either… or*, etc.), or verbs of hedging (*suggest*, *suspect*, *indicate*, *suppose*, *seem*, *appear*, etc.). The following examples from the BioScope corpus [2] illustrate how cue words are annotated in the Shared Task training data:

(1) {The specific role of the chromodomain is <**unknown**>} but chromodomain swapping experiments in Drosophila {<**suggest**> that they {<**might**> be protein interaction modules}} [18].

(2) These data {<**indicate that**> IL-10 and IL-4 inhibit cytokine production by different mechanisms}.

(3) Whereas a background set of promoter regions is easy to identify, it is {<**not clear**> how to define a reasonable genomic sample of enhancers}.

(4) {This domain is <**predicted**> to function analogously in Transib transposons}.

In the examples above, hedge cues are shown using angle brackets, with braces corresponding to their annotated scopes. Moreover, the training data also annotates an entire sentence as *uncertain* if it contains a hedge cue, and it is the prediction of this sentence labeling that is required for Task 1.

Judging by the examples above, it might at first seem that the hedge cues can be identified merely by consulting a pre-compiled list. However, most, if not all, words that can function as hedge cues can also occur as non-cues. More than 85% of the hedge cues observed in the BioScope corpus also have non-cue occurrences. As an example, consider the four different usages of *appear* in the sentences below. A hedge detection system needs to correctly discriminate between its use as a hedge cue in Examples 5-6, and as a non-cue in Examples 7-8.

(5) Furthermore, these cell lines {<**appear**> resistant to lysis by natural killer (NK) cells}.

(6) In 5 patients the granulocytes {<**appeared**> polyclonal} and in 1 patient unilateral X inactivation was observed in both granulocytes and T cells.

(7) If an organism has many readthrough proteins, proteins from the organism will frequently **appear** in the 273 clusters.

(8) The effect **appeared** within 30 min and returned to basal levels after 2 h.

The approach presented in this paper extends on that of Velldal et al. [3], where a maximum entropy (MaxEnt) classifier is applied to automatically detect cue words, subsequently labeling sentences as uncertain if they are found to contain a cue. Furthermore, in the system of Velldal et al. [3], the resolution of the in-sentence scopes of identified cues, as required for Task 2, is determined by a set of manually crafted rules operating on dependency representations. Readers that are interested in more details on this set of scope rules are referred to Øvrelid et al. [4]. The focus of the present paper, however, is to present a new and simplified approach to the classification problem relevant for solving Task 1, and also partially Task 2, viz. the identification of hedge cues.

### Related work

The top-ranked system for Task 1 in the official CoNLL 2010 Shared Task evaluation, described by Tang et al. [5], approaches cue identification as a *sequence labeling problem.* Similarly to Morante et al. [6], Tang et al. [5] set out to label tokens according to a BIO-scheme, i.e. indicating whether they are at the Beginning, Inside, or Outside of a hedge cue. Tang et al. [5] train both a Conditional Random Field (CRF) sequence classifier and an SVM-based Hidden Markov Model (HMM), finally combining the predictions of both models in a second CRF.

In terms of the overall approach, i.e. viewing the problem as a sequence labeling task, Tang et al. [5] are actually representative of the majority of the ST participants for Task 1 [1], including the top three performers on the official held-out data. As noted by Farkas et al. [1], the remaining systems approached the task either as a word-by-word *token classification problem*, or directly as a *sentence classification problem.* Examples of the former are the systems of Velldal et al. [3] and Vlachos et al. [7], sharing the 4th rank position (out of 24 submitted systems) for Task 1. In both the sequence labeling and token classification approaches, a sentence is labeled as uncertain if it contains a word labeled as a cue. In contrast, the sentence classification approaches instead try to label sentences directly, typically using Bag-of-Words (BoW) features. In terms of the official Task 1 evaluation, the sentence classifiers tended to achieve a somewhat lower relative rank.

### Our approach

The approach presented in this paper extends on the token classification approach described by Velldal et al. [3], but is set within the framework of Support Vector Machine (SVM) classification [8] instead of MaxEnt. Moreover, rather than attempting to classify *all* tokens, we show how better results can be obtained by instead approaching the task as a *disambiguation problem*, restricting our attention to only those tokens whose base forms have previously been observed as hedge cues. Reformulating the problem in this way simplifies the classification task tremendously, reducing the number of examples that need to be considered, and thereby also trimming down the relevant feature space to a much more manageable size. The resulting feature space is still rather huge, however, and we show how the method of *random indexing* (RI) can be used to further reduce the dimensionality by several orders of magnitude while still preserving performance. For the problem of sentence-level uncertainty prediction, the classifiers developed in this paper achieves the best published results so far on the CoNLL-10 Shared Task data (to the best of our knowledge).

## Methods

In this section we first give a brief description of the CoNLL-10 Shared Task data sets, including the relevant preprocessing applied for our experiments. We then turn to develop an initial SVM-based hedge cue classifier along the lines of Velldal et al. [3], also giving some more details about the evaluation measures and the feature templates that we use. For a given sentence, the classifier considers each word in turn, labeling it as a *cue* or a *non-cue*. We will refer to this mode of cue classification as performing *word-by-word classification* (WbW). Later we go on to show how better results can be obtained by reformulating the task as a *disambiguation problem* restricted to only those tokens whose base forms have previously been observed as hedge cues, instead of performing WbW classification across all tokens. Note that, in both set-ups, any sentence found to contain a cue is subsequently labeled as *uncertain*. Finally, we describe the framework of *random indexing* (RI)—a dimensionality reduction technique that can be viewed as sparse random projections. Using RI, we show that our very high-dimensional feature space can be compressed by two orders of magnitude without sacrificing classifier performance.

Note that, while preliminary results for all models are presented for the development data throughout the paper, the performance of all models is ultimately compared on the official Shared Task held-out data in the *Results and discussion* section.

### Data sets and preprocessing

The training data for the CoNLL 2010 Shared Task is taken from the BioScope corpus [2] and consists of 14,541 sentences (or other root-level utterances) from biomedical abstracts and articles. Some basic descriptive statistics for the data sets are provided in Table 1. We see that roughly 18% of the sentences are annotated as uncertain. The BioScope corpus also provides annotation for hedge cues as well as their scope. Out of a total of 378,213 tokens, 3,838 are annotated as being part of a hedge cue. As can be seen, the total number of cues is somewhat lower (3,327), due to the fact that some tokens are part of the same cue, so-called multi-word cues (448 in total), such as *indicate that* in Example 2 above.

For evaluation purposes, the task organizers provided newly annotated biomedical articles, comprising 5,003 additional utterances, of which 790 are annotated as hedged (see Table 1). The data contains a total of 1,033 cues, of which 87 are multi-word cues spanning multiple tokens, comprising 1,148 cue tokens altogether.

**Table 1:** **The Shared Task data sets** The top three rows lists the properties of the training data, separately detailing its two components—biomedical abstracts and full articles. The bottom row summarizes the official held-out test data (articles only). Token counts are based on the tokenizer described above.

Data Set	Sentences	Hedged Sentences	Cues	Multi-Word Cues	Tokens	Cue Tokens
**Abstracts**	11,871	2,101	2,659	364	309,634	3,056
**Articles**	2,670	519	668	84	68,579	782
**Total (train)**	14,541	2,620	3,327	448	378,213	3,838
**Held-Out**	5,003	790	1,033	87	138,276	1,148

#### Tokenization

The GENIA tagger [9] takes an important role in our preprocessing set-up, as it is specifically tuned for biomedical text. Nevertheless, its rules for tokenization appear to not always be optimally adapted for the BioScope corpus. (For example, GENIA unconditionally introduces token boundaries for some punctuation marks that can also occur token-internally.) Our preprocessing pipeline therefore employs a home-grown, cascaded finite-state tokenizer (adapted from the open-source English Resource Grammar; [10]), which aims to implement the tokenization decisions made in the Penn Treebank [11]—much like GENIA, in principle—but properly treating certain corner cases found in the BioScope data.

#### PoS tagging and lemmatization

For part-of-speech (PoS) tagging and lemmatization, we combine GENIA and TnT [12], which operates on pre-tokenized inputs but in its default model is trained on financial news from the Penn Treebank. Our general goal here is to take advantage of the higher PoS accuracy provided by GENIA in the biomedical domain, while using our improved tokenization.

For the vast majority of tokens, we use GENIA PoS tags and base forms (i.e. lemmas). However, GENIA does not make a PoS distinction between proper and common nouns, as in the Penn Treebank, and hence we give precedence to TnT outputs for tokens tagged as nominal by both taggers.

### Word-by-word cue classification

This subsection describes our initial WbW cue classifier. For a given sentence, a binary SVM-classifier labels each word as a *cue* or a *non-cue*, subsequently labeling the entire sentence as *uncertain* if it is found to contain a cue.

#### Defining the training instances

As annotated in the training data, it is possible for a hedge cue to span multiple tokens, e.g. as in *whether or not*. The majority of the multi-word cues in the training data are very infrequent, however, most occurring only once, and the classifier itself is not sensitive to the notion of multi-word cues. A given word token is considered a cue as long as it falls within the span of a cue annotation.

As presented to the learner, a given token *w_i_* is represented as a feature vector . Each dimension *f*_ij_ represents a feature function which can encode arbitrary properties of *w_i_*. The particular features we are using are described below. Each training example can be thought of as a pair of a feature vector and a label, . If *w_i_* is a cue we have *y_i_*=+1, while for non-cues the label is –1. For estimating the actual SVM classifier for predicting the labels on unseen examples we use the SVM*^light^* toolkit [13].

#### Evaluation measures

We will be reporting precision, recall and F_1_ for two different levels of evaluation; the *sentence-level* and the *token-level.* While the token-level scores indicate how well the classifiers succeed in identifying individual cue words, the sentence-level scores are what actually correspond to Task 1, i.e. correctly identifying whether a sentence contains uncertainty or not. Moreover, when comparing the scores of different classifiers we will be applying a two-tailed *sign-test* for assessing the statistical significance of any differences. This is a standard non-parametric test for paired samples, which in our setting considers how often the classifier decisions of two given models differ (with respect to either individual tokens or entire sentences). We will assume a standard significance level of α=0.05.

#### Feature templates

In the Shared Task system description paper of Velldal et al. [3], results are reported for MaxEnt cue classifiers using a wide variety of feature types of both surface-oriented and syntactic nature. For the latter, Velldal et al. [3] define a range of syntactic and dependency-based features extracted from parses produced by the MaltParser [14,15] and the XLE [16], recording information about dependency relations, subcategorization frames, etc. However, it turned out that the simpler lexical and surface-oriented features were sufficient for the identification of hedge cues.

Drawing on the observation above, the classifiers trained in this paper are only based on simple sequence-oriented *n*-gram features collected for PoS-tags, lemmas and surface forms. For all these types of features we record neighbors for up to 3 positions left/right of the focus word. For increased generality, all these *n*-gram features also include non-lexicalized variants, i.e. excluding the focus word itself.

Instantiating all feature templates described above for the BioScope training data, using the maximal span for all *n*-grams (*n*=4, i.e. including up to 3 neighbors), we end up with a total of more than 6,500,000 unique feature types. However, after testing different feature configurations, it turns out that the best performing model only uses a small subset of this feature pool. The configuration we will be using throughout this paper includes; *n*-grams over base forms ±3 positions of the focus word; *n*-grams over surface forms up to +2 positions only; and PoS of the focus word. This results in a set of roughly 2,630,000 feature types. In addition to reporting classifier performance for this feature configuration, we also provide results for a baseline model using only *unigram* features over surface forms. The behavior of this classifier is similar to what we would expect from simply compiling a list of cue words from the training data, based on the majority usage of each word as cue or non-cue.

#### Preliminary results

As shown in Table 2, after averaging results from 10-fold cross-validation on the training data, the baseline cue classifier described above (shown as ) achieves a sentence-level F_1_ of 88.69 and a token-level F_1_ of 79.59. In comparison, the classifier using all the available *n*-gram features (*C_WbW_*) achieves F-scores of 91.19 and 87.80 on the sentence-level and token-level, respectively. We see that the improvement in performance compared to the baseline is most pronounced on the token-level, but the differences in scores for both levels are found to be statistically significant when applying a two-tailed sign-test as described above (giving p-values that approaches zero; p≈1.5× *e*^–11^).

### Reformulating the classification problem

An error analysis of our initial WbW classifier revealed that it is not able to generalize to new hedge cues beyond those that have already been observed during training. Even after adding the non-lexicalized variants of all feature types (i.e. making features more general by not recording the focus word itself), the classifier still fails to identify any unseen hedge cues whose base form did not occur as a cue in the training material. On the other hand, only very few of the test cues are actually unseen (≈1.5%), meaning that the set of cue words might reasonably be treated as a near-closed class (at least for the biomedical data considered in this study). As a consequence of these observations, we here reformulate the problem as follows. Instead of approaching the task as a classification problem defined for all words, we only consider words that have a base form observed as a hedge cue in the training material. In effect, any word whose base form has never been observed as a cue in the training data is automatically considered to be a non-cue when testing. Part of the rationale here is that, while it seems reasonable to assume that any word occurring as a cue can also occur as a non-cue, the converse is less likely. (As noted in the introduction, more than 85% of the observed cue lemmas also have non-cue occurrences in the training data.)

While the training data contains a total of approximately 17,600 unique base forms (given the preprocessing outlined above), only 143 of these ever occur as hedge cues. By restricting the classifier to only this subset, we manage to simplify the classification problem tremendously, but without any loss in performance.

Note that, although we will approach the task as a disambiguation problem, it is not feasible to train separate classifiers for each individual base form. The frequency distribution of the cue words in the training material is very skewed with most cues being very rare—many occurring as a cue only once (≈ 40%). (Most of these words also have many additional occurrences in the training data as non-cues, however.) For the majority of the cue words then, it seems we can not hope to gather enough reliable information to train individual classifiers. Instead, we want to be able to draw on information from the more frequently occurring cues also when classifying or disambiguating the less frequent ones. Consequently, we still train a single global classifier as for the original WbW set-up. However, as the disambiguation classifier still only needs to consider a small subset of the number of words considered by the full WbW classifier, the number of instantiated feature types is, of course, greatly reduced.

For the full WbW classification, the number of training examples is 378,213. Using the feature configuration described above, this generates a total of roughly 2,630,000 feature types. For the disambiguation model, using the same feature configuration, the number of instantiated feature types is reduced to just below 670,000, as generated for 94,155 training examples.

#### Preliminary results

Running the new disambiguation classifier by 10-fold cross validation on the training data, we find that it has substantially better recall than the original WbW classifier. The results are shown in the row *C_Disamb_* in Table 2. Across all levels of evaluation the *C_Disamb_* model achieves a boost in F_1_ compared to *C_WbW_*. However, when applying a two-tailed sign-test, considering differences in classifier decisions on both the sentence-level (*p* ≈ 0.057) and token-level (*p* ≈ 0.00025), only the latter differences are found to be significant (at *α* = 0.05).

#### The effect of data size

**Figure 1:**
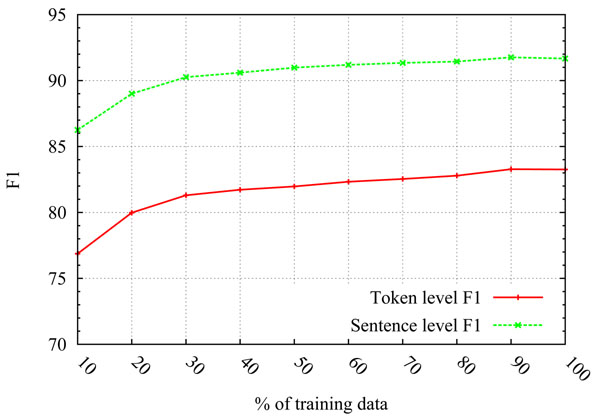
**Learning curves** shows the effect of incrementally including a larger percentage of the training data into the 10-fold cycles for the disambiguation classifier. The learning curves plot the effect for both token- and sentence-level F-scores.

Given how the disambiguation classifier treats the set of cue words as a closed class, a reasonable concern is its sensitivity to the size of the training set. In order to assess this effect, we computed learning curves showing how classifier performance changes as more training examples are added. Starting with only 10% of the training data included in each fold of a 10-fold cycle, Figure 1 shows the effect on both token-level and sentence-level F-scores as we incrementally include larger portions of the available training data, up until we reach 100%. We see that classifier performance is steadily improving as more training data is included, but the curves seem to gradually flatten out as we approach the 90% mark, at which point the performance seems to have already reached its peak for this particular data set.

Looking closer at distribution of error types (considering the 10-fold run that used 100% of the data), we find that roughly 75% of the errors are false negatives, leaving 25% false positives. However, rather than being caused by legitimate cue words being filtered out during training, the false negatives mostly pertain to a handful of high frequency words that are also highly ambiguous. For example, *or* alone comprises almost 25% of the total number of false negatives, and *can* comprises another 10%. Looking at the distribution of these words in the training data, it is easy to see how they can represent a challenge for the learner: While the total number of occurrences of *or* and *can* is 1215 and 506 respectively, they are both annotated as non-cues 77% of the time.

### Sparse random indexing

As mentioned above, each training example is represented by a *d*-dimensional feature vector . Given *n* examples and *d* features, the feature vectors can be thought of as rows in a matrix F ∈ ℜ^n×d^. One potential problem with using a vector-based numerical encoding of local context features, is that the dimensionality of the feature space grows very rapidly with the number of training examples. Using local features, e.g. context windows recording properties such as direction and distance, the number of unique features grows much faster than when using, say, BoW features. In order to make the vector encoding scalable, we would like to somehow be able to put a bound on the number of dimensions.

As mentioned above, even after simplifying the classification problem, our input feature space is still rather huge, totaling roughly 670,000 feature types. Given that the number of training examples is only around *n* ≈ 95,000 we have that *d*≫*n*, and whenever we want to add more feature templates or add more training data, this imbalance will only become more pronounced. It is also likely that many of the *n*-gram features in our model will not be relevant for the classification of new data points. The combination of many irrelevant features, and few training examples compared to the number of features, makes the learner prone to overfitting.

In previous attempts to reduce the feature space, we have applied several *feature selection* schemes, such *as filtering* on the correlation coefficient between a feature and a class label, or using simple frequency cutoffs. Although such methods are effective in reducing the number of features, they typically do so at the expense of classifier performance. Due to both data sparseness and the likelihood of many features being only locally relevant, it is difficult to reliably assess the relevance of the input features, and we risk filtering out many relevant features as well. Using simple filtering methods, we did not manage to considerably reduce the number of features without also significantly reducing the performance of the classifier. Although better results can be expected by using so-called *wrapper methods*[17] instead, this is not computationally feasible for large feature sets.

As an alternative to such feature selection methods, we here report on experiments with a technique known as *random indexing* (RI). This allows us to drastically compress the feature space without explicitly throwing out any features.

The technique of random indexing was initially introduced by Kanerva et al. [18] for modeling the semantic similarity of words by their distribution in text. (Readers are referred to [19] for a good introduction to random indexing.) Actually RI forms part of a larger family of dimension reduction techniques based on *random projections.* Such methods typically work by multiplying the feature matrix *F* ∈ ℜ*^n^*^×^*^d^* by a random matrix *R* ∈ ℜ*^d^*^×^*^k^*, where *k* ≪ *d*, thereby reducing the number of dimensions from *d* to *k*:(1)

Given that *k* is sufficiently high, the Johnson-Lindenstrauss lemma [20] tells us that the pairwise distances (and thereby separability) in *F* can be preserved with high probability within the lower-dimensional space *G*[21]. While the only condition on the entries of *R* is that they are i.i.d. with zero mean, they are typically also specified to have unit variance [21].

One particular advantage of the random indexing approach is that the full *n* × *d* feature matrix *F* does not need to be explicitly computed. The method constructs the representation of the data in *G* by *incrementally accumulating* so-called *index vectors* assigned to each of the *d* features [22]. The process can be described by the following two simple steps:

- When a new feature is instantiated, it is assigned a randomly generated vector of a fixed dimensionality *k*, consisting of a small number of –1s and +1s (the remaining elements being 0). This is then the so-called *index vector* of the feature. (The index of the *i*th feature corresponds to the *i*th row of *R.*)

*-* The vector representing a given training example (the *j*th row of *G* represents the *j*th example) is then constructed by simply summing the random index vectors of its features.

Note that, although we want to have *k* ≪ *d*, we still operate in relatively high-dimensional space (with *k* being on the order of thousands). As demonstrated in [23], high-dimensional vectors having random directions are very likely to be close to orthogonal, and the approximation to *F* will generally be better the higher we set *k*[19].

Finally, it is worth noting that RI has traditionally been applied on the *type level*, with the purpose of accumulating context vectors that represent the distributional profiles of words in a semantic space model [19]. Here, on the other hand, we apply it on the *instance level* and as a general means of compressing the feature space of a learning problem.

#### Tuning the random indexing

**Figure 2:**
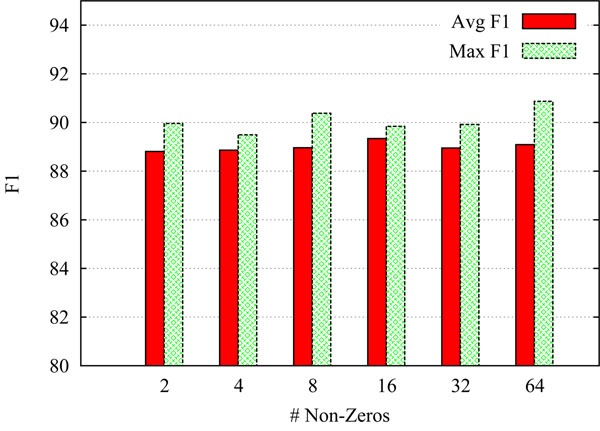
**Varying the number of non-zeros in the index vectors** shows the effect of varying the number of non-zero elements in the random index vectors, while keeping the dimensionality fixed at *k*=5,000. The plot shows averaged and maximum sentence-level F_1_ across 5 different runs for each setting (using different random initializations of the index vectors), testing on 1/10th of the training data. For reference, the last column shows the result for using the original non-projected feature space.

Regarding the *ratio of non-zero elements*, the literature on random projections contains a wide range of suggestions as to how the entries of the random matrix *R* should be initialized. In the context of random indexing, Sahlgren et al. [22] set approximately 1% of the entries in each index to +1 or –1. It is worth bearing in mind, however, that the computational complexity of dot-product operations (as used extensively by the SVM learner) depend not only on the number of dimensions itself, but on the number of non-zero elements. We therefore want to take care to avoid ending up with a reduced space that is much more dense. Nevertheless, the appeal of using a random projection technique is in our case more related to its potential as a feature extraction step, and less to its potential for speeding up computations and reducing memory load, as the original feature vectors are already very sparse. After experimenting with different parametrizations, it seems that the classifier performance on our data sets are fairly stable with respect to varying the ratio of non-zeros. Moreover, we find that the non-zero entries can be very sparsely distributed, e.g. ≈ 0.05-0.2%, without much loss in classifier performance. Figure 2 shows the effect of varying the ratio of non-zero elements while keeping the dimensionality fixed (at *k*=5,000), always assigning an equal number of +1s and -1s (giving zero mean and unit variance). For each parametrization we perform a batch of 5 experiments using different random initializations of the index vectors. The scores shown in Figure 2 are the average and maximum F_1_ within each batch. As can be seen, with index vectors of 5,000 elements, it seems that using 8 non-zero entries (corresponding to a ratio of 0.16%) strikes a reasonable balance between index density and performance for our data set.

**Figure 3:**
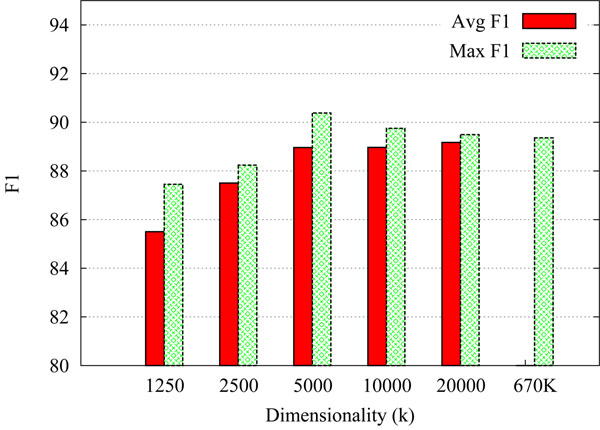
**Varying the dimensionality of the index vectors** shows the effect of varying the dimensionality (*k*) of the random index vectors. The ratio of non-zero elements is kept fixed, varying from 2 (for *k*=1,250) to 32 (for *k*=20,000). The plot shows averaged and maximum sentence-level F_1_ across 5 different runs for each setting (using different random initializations of the index vectors), testing on 1/10th of the training data. For reference, the last column shows the result for using the original non-projected feature space of 670,000 dimensions.

As expected, we do, however, see a clear deterioration of classifier accuracy if the *dimensionality* of the index vectors is set very low. Figure 3 shows the effect of varying the dimensionality *k* of the index vectors, while fixing the ratio of non-zero entries per vector to 0.16%. Again we perform batches of 5 experiments for each value of *k*, reporting the average and maximum within each batch. For our cue classification data, the positive effect of increasing *k* seems to flatten out at around *k*=5,000. When considering the standard deviation of scores within each batch, however, the variability of the results seems to steadily decrease as *k* increases. For example, while we find σ=1 .34 for the set of runs using *k*=1,250, we find σ=0.29 for *k*=20,000.

When looking at the *maximum scores* shown in Figure 3, one of the runs using *k*=5,000 turns out to have the peak performance, achieving a (sentence-level) F_1_ of 90.38. Not only does it score higher than any of the other RI-runs with *k*>5,000, it also outperforms the original *C_Disamb_* model, which achieves an F_1_ of 89.36 for the same single “fold” (the models in Figure 3 are tested using 1/10th of the training material).

In our experience, although the random projection provided by the RI vectors only represents an approximation to the original input space, it still appears to preserve a lot more information than feature selection based on filtering methods.

#### Preliminary results

The bottom row of Table 2 shows the results of applying an SVM-classifier by full 10-fold cross-validation over the training set using the same random index assignments that yielded the maximum F_1_ in Figure 3 for *k*=5,000 (with eight randomly set non-zeros in each index). We see that the performance of  is actually slightly lower than for *C_Disamb_*. Applying the sign-test to the differences in classifier decisions we find *p*≈0.041 for the sentence-level and *p*≈0.0001 for the token-level. It should be pointed out that we have not yet tried tuning the random indexing by multiple runs of full 10-fold cross-validation on the training data, which would be expected to improve these results. Given the fact that the effective feature space for the classifier is reduced from 670,000 to just 5,000 dimensions, we find it notable that the  model achieves comparable results, with only preliminary tuning.

**Table 2:** **Development results** Development results for the various hedge classifiers tested by 10-fold cross-validation on the biomedical abstracts and articles in the training data.

	Sentence Level			Token Level		
Model	Prec	Rec	F1	Prec	Rec	F1
	91.01	86.53	88.69	90.60	71.03	79.59
*C_WbW_*	94.31	88.30	91.19	94.67	81.89	87.80
*C_Disamb_*	93.64	89.68	91.60	94.01	83.55	88.45
	93.78	88.45	91.03	94.05	81.97	87.58

Another important observation is that the *complexity* of the resulting SVM in terms of the number of support vectors (SVs), is considerably reduced for the RI-model: While the number of SVs for *C_Disamb_* averages just below 8% of the training examples, this is reduced to 4% for  (using the SVM*^light^* default settings). In addition to halving the number of SVs, as well as reducing the features by two orders of magnitude, the upper bound on the VC-dimension (as estimated by SVM*^light^*) is also reduced by 12%. It is also worth noting that the run-time differences for estimating the SVM on the original input space and the reduced (but slightly denser) feature space, are negligible (≈ 5 CPU-secs. more for the RI-model when re-training on the full training set).

## Results and discussion

### Held-out testing

Table 3 presents the final results for the various classifiers developed in this paper, testing them on the biomedical articles of the CoNLL 2010 Shared Task held-out test set (see Table 1). In addition to the evaluation results for our own classifiers, Table 3 also includes the official test results for the system described by Tang et al. [5]. The sequence classifier developed by Tang et al. [5], combining a CRF classifier and a large-margin HMM model, obtained the best results for the official ST evaluation for Task 1 (i.e. sentence-level uncertainty detection).

As seen from Table 3, all of our SVM classifiers *C_WbW_*, *C_Disamb_*, and  achieve a higher sentence-level F_1_ than the system of Tang et al. [5] (though it is unknown whether the differences are statistically significant). We also note that our reformulation of the cue classification task as a disambiguation problem leads to better performance also on the held-out data, with *C_Disamb_* performing slightly better than *C_WbW_* across both evaluation levels. Interestingly, the best performer of them all proves to be the random indexing model  even though this model was not the top-performer on the training data. One possible explanation for the strong held-out performance of  is that the reduced complexity of this classifier (as discussed in the previous subsection) has made it less prone to overfitting, leading to better generalization performance on new data. Applying the sign-test as described above to the classifier decisions of  we find statistically significant differences with respect to *C_WbW_* (*p* gets rounded to zero for both the sentence- and token-level) but not with respect to *C_Disamb_* (*p*≈0.68 for the sentence-level and *p*≈0.34 for the token-level). Nonetheless, the encouraging results of the  model on the held-out data means that further tuning of the RI configuration on the training data will be a priority for future experiments. It is also worth noting that many of the systems participating in the ST challenge used fairly complex and resource-heavy feature types, being sensitive to document structure, grammatical relations, etc. [1]. The fact that comparable or better results can be obtained using a relatively simple approach as demonstrated in this paper—with low cost in terms of both computation and external resources—might lower the bar for employing a hedge detection component in an actual IE system.

**Table 3:** **Held-out results** Final test results for the various hedge classifiers on the Shared Task test data.

	Sentence Level			Token Level		
Model	Prec	Rec	F1	Prec	Rec	F1
	77.54	81.27	79.36	75.89	66.90	71.11
*C_WbW_*	89.02	84.18	86.53	87.58	74.30	80.40
*C_Disamb_*	87.37	85.82	86.59	85.92	76.57	**80.98**
	88.83	84.56	**86.64**	86.65	74.65	80.21
Tang	85.03	87.72	86.36	–	–	–

Finally, we also observe that our simple unigram baseline classifier proves to be surprisingly competitive. In fact, comparing its Task 1 F_1_ to those of the official ST evaluation, it actually outranks 7 of the 24 submitted systems. For future work, we plan to investigate the use of *hash functions* for providing the randomized mapping from input features into the positions in the lower-dimensional index vectors. This would eliminate the need to store the set of random index vectors, represented by *R* ∈ ℜ*^d×k^* in Equation 9.

## Conclusions

This paper has presented the incremental development of uncertainty classifiers for detecting hedging in biomedical text—the topic of the CoNLL 2010 Shared Task. Using simple *n*-gram features over words, lemmas and PoS-tags, we first develop a (linear) SVM cue classifier that outperforms the top ranked system for Task 1 in the official Shared Task evaluation (i.e. sentence-level uncertainty detection). We then show how the original classification task can be greatly simplified by viewing it as a disambiguation task restricted to only those words that have previously been observed as hedge cues. Operating in a smaller (though still fairly large) feature space, this second classifier achieves even better results. Finally, we apply the method of random indexing, further reducing the dimensionality of the feature space by two orders of magnitude. This final classifier—combining an SVM-based disambiguation model with random indexing—is our best performer, achieving a sentence-level F_1_ of 86.64 on the CoNLL 2010 Shared Task held-out data.

## Competing interests

The author declares having no competing interests.
